# Strategies for precision vagus neuromodulation

**DOI:** 10.1186/s42234-022-00091-1

**Published:** 2022-05-30

**Authors:** Umair Ahmed, Yao-Chuan Chang, Stefanos Zafeiropoulos, Zeinab Nassrallah, Larry Miller, Stavros Zanos

**Affiliations:** 1 Institute of Bioelectronic Medicine, Feinstein Institutes for Medical Research, Manhasset, New York, USA; 2 Donald and Barbara Zucker School of Medicine at Hofstra/Northwell, Hempstead, New York, USA

**Keywords:** Vagus fibers, Fascicles, Branches, Selective vagus nerve stimulation, Neuromodulation, Noninvasive, Ultrasound, Bioelectronic medicine

## Abstract

The vagus nerve is involved in the autonomic regulation of physiological homeostasis, through vast innervation of cervical, thoracic and abdominal visceral organs. Stimulation of the vagus with bioelectronic devices represents a therapeutic opportunity for several disorders implicating the autonomic nervous system and affecting different organs. During clinical translation, vagus stimulation therapies may benefit from a precision medicine approach, in which stimulation accommodates individual variability due to nerve anatomy, nerve-electrode interface or disease state and aims at eliciting therapeutic effects in targeted organs, while minimally affecting non-targeted organs. In this review, we discuss the anatomical and physiological basis for precision neuromodulation of the vagus at the level of nerve fibers, fascicles, branches and innervated organs. We then discuss different strategies for precision vagus neuromodulation, including fascicle- or fiber-selective cervical vagus nerve stimulation, stimulation of vagal branches near the end-organs, and ultrasound stimulation of vagus terminals at the end-organs themselves. Finally, we summarize targets for vagus neuromodulation in neurological, cardiovascular and gastrointestinal disorders and suggest potential precision neuromodulation strategies that could form the basis for effective and safe therapies.

## Background

The vagus is the tenth and longest cranial nerve which brings the brain and internal organs in bidirectional communication (Câmara and Griessenauer 2015). It originates in the brainstem and innervates most visceral structures, including the heart, lungs, and gastrointestinal system (Câmara and Griessenauer 2015), regulating physiological homeostasis via various autonomic reflexes. The vagus nerve is easily surgically accessible at the cervical level (Giordano et al. 2017), making it a preferred target for autonomic neuromodulation therapies. However, the micro-anatomical structure of the vagus nerve trunk at the cervical level, especially in humans, is relatively complex and varies between individuals (Pelot et al. 2020). It includes several different types and sub-types of nerve fibers, according to their morphological, physiological and functional properties (Agostoni et al. 1957). Different fiber types convey distinct afferent and efferent signals and mediate specific visceral-sensory and visceral-motor functions (Y. C. Chang et al. 2020).

The safety and efficacy profile of vagus stimulation, determined largely by the nerve fiber populations activated by stimuli, depend on which vagus-innervated organ and vagus-mediated function is being considered. Even though the increasing dose of stimulation generally improves efficacy, the optimal dose for a targeted organ might be outside the therapeutic range for other, non-targeted, organs causing adverse effects and sometimes discontinuation of therapy. For example, after it was determined that vagus stimulation is an effective therapy in preclinical studies of heart failure (Sabbah 2011; Sabbah et al. 2007; Zhang et al. 2009), cervical VNS was tested in clinical trials (De Ferrari et al. 2011; Gold et al. 2016; Zannad et al. 2015). At the stimulation dose that achieved the desired effect in the heart – cardioinhibition and modest bradycardia –most patients reported undesired effects related to vagal innervation of the larynx, including throat pain, cough, hoarse voice, as well as nausea, and vomiting, which were considered to be significant safety issues (De Ferrari et al. 2011; Premchand et al. 2014). Other studies tested lower doses of VNS to maintain a long-term safety profile across organs and subjects, but at that dose, no improvements in efficacy were observed (De Ferrari et al. 2017; Gold et al. 2016; Zannad et al. 2015). Without a strategy to provide precision neuromodulation to the vagus and optimize safety and efficacy profiles depending on which organ and function is targeted, future therapeutic uses of vagus stimulation may be hindered by similar undesired effects.

In the first part of this review, we describe anatomical and physiological features of the vagus that provide the basis for precision vagus neuromodulation. Those features are discussed at several levels, from individual nerve fibers and fascicles to vagus branches and the organs they innervate. In the second part, we discuss several strategies that leverage the anatomical and functional organization of the vagus to deliver precision neuromodulation, including fascicle- or fiber-selective cervical vagus nerve stimulation, stimulation of vagal branches near the end-organs, and ultrasound stimulation of vagus terminals at the end-organs themselves. Finally, we summarize targets for vagus neuromodulation in neurological, cardiovascular and gastrointestinal disorders and suggest potential precision neuromodulation strategies that could form the basis for effective and safe therapies.

## Functional anatomy of the vagus

### Vagal branches and organs they innervate

The vagus nerve gives rise to several branches that innervate visceral structures (Câmara and Griessenauer 2015). After leaving the skull through the jugular foramen, the vagus nerve enters the cervical region, where the cervical vagus nerve (cVN) is a prime target for neuromodulation in several diseases (Berthoud and Neuhuber 2000a, 2000b). Electrode placement at this level requires a minor surgery with minimal surgical risks. The first two branches leaving the vagus at the level of the jugular ganglion are the auricular and meningeal branches, providing sensory innervation to the skin of the external acoustic meatus and the dura of the posterior cranial fossa, respectively (Berthoud and Neuhuber 2000a, 2000b). At the cervical level, it gives rise to the pharyngeal nerve, and superior laryngeal nerve (Henry Gray 2020). The pharyngeal nerve, branching from the vagus nerve just distal to the nodose ganglion, contains both sensory and motor fibers. It carries sensory information from the epiglottis and root of the tongue. It is the primary motor nerve of the pharynx and palate muscles (Henry Gray 2020). The superior laryngeal nerve branches from the vagus nerve near carotid bifurcation and divides into internal and external laryngeal nerves near the hyoid bone. The internal laryngeal nerve supplies the mucosa between the epiglottis and vocal folds, whereas the external laryngeal nerve supplies cricothyroid muscle (Henry Gray 2020). The recurrent laryngeal nerve (also known as inferior laryngeal nerve) follows a different route on the right and left sides. The right recurrent laryngeal nerve branches off in the superior mediastinum, loops around the right subclavian artery and ascends to the larynx in the tracheoesophageal groove on the right. The left recurrent laryngeal nerve also branches off in the superior mediastinum at the level of the aortic arch. It then loops around the aortic arch and ascends to the larynx in the tracheoesophageal groove on the left. The recurrent laryngeal nerve provides motor innervation to all intrinsic laryngeal muscles, and sensory innervation to the laryngeal mucosa inferior to the vocal folds (Henry Gray 2020).

The vagus nerve provides parasympathetic stimulation to thoracic viscera and visceral afferent fibers relaying reflexive sensations such as stretch. Cardiac branches that arise bilaterally from the cervical and thoracic vagal nerves contribute to cardiac innervation (Henry Gray 2020; Kawashima 2005). These nerves contain preganglionic parasympathetic fibers that join sympathetic cardiac nerves to form the deep and superficial cardiac plexuses at the base of the heart (Kawashima 2005). The cardiac plexuses innervate the sinoatrial and atrioventricular nodes which have a cardioinhibitory function through vagal fibers and cardioexcitatory function through sympathetic fibers (Henry Gray 2020). The vagus also innervates the lungs and bronchial tree via pulmonary branches which arise near the tracheal bifurcation. Similar to the pattern of cardiac innervation, the pulmonary branches of the vagus nerve, along with sympathetic fibers, contribute to the anterior and posterior pulmonary plexuses. The pulmonary plexuses control smooth muscle tone, glandular secretion, and vascular tone (Henry Gray 2020; Moore et al. 2017). Pulmonary branches provide sensory innervation to chemoreceptors and stretch receptors by innervating the pulmonary vasculature. (Chang et al. 2015; Henry Gray 2020; Moore et al. 2017).

After coursing posterior to the root of the lungs, the left and right vagus nerves, along with sympathetic fibers, contribute to the esophageal plexus. This plexus controls the esophageal smooth muscle and glands via the vagal fibers, and the esophageal vasculature via the sympathetic fibers. Sensory fibers from the muscular and mucosal layers are relayed via the vagus nerve (Henry Gray 2020; Hornby and Abrahams 2000; Hudson and Cummings 1985).

From the esophageal plexus arise two vagus trunks, routed anteriorly (ventral) and posteriorly (dorsal) to the esophagus, known as anterior and posterior trunks of the vagus (Ellis 1997). The anterior trunk of the vagus contains fibers predominantly from the left vagus, whereas the posterior trunk predominantly from the right vagus (Ellis 1997). Both enter the abdomen through the esophageal hiatus at the level of the tenth thoracic vertebra. The anterior and posterior trunks innervate the liver, spleen, pancreas, and gastrointestinal tract as far as the splenic flexure (Manter 1975). These organs are innervated either through the direct branch of the vagus trunks or indirectly through celiac, superior mesenteric, and renal plexuses (Ellis 1997; Monkhouse 2005). Vagal stimulation produces motility and glandular secretion in the gut; however, the vagus nerve consists mainly of afferent fibers relaying reflexive sensory information from the gut to the brain (Agostoni et al. 1957; Henry Gray 2020).

The liver is innervated by the hepatic branch from the anterior vagal trunk (Berthoud and Neuhuber 2000a, 2000b). The vagal fibers are mostly present at the porta hepatis, which consists of the portal vein, hepatic artery, and bile duct (Berthoud et al. 1992; Berthoud and Neuhuber 2000a, 2000b). However, some studies have also suggested vagal innervation at the liver parenchyma (Forssmann and Ito 1977; Metz and Forssmann 1980; Tiniakos et al. 1996). The stomach and small intestines are also supplied by the vagus nerve through hepatic, gastric, and intestinal branches. The vagal innervations are present in the submucosal and muscular layers in the digestive tract, also known as meissner and myenteric plexus, respectively (Berthoud and Neuhuber 2000a, 2000b). The prominent supply in the stomach is from the myenteric plexus innervating primarily mechano-sensors (Berthoud and Neuhuber 2000a, 2000b; Berthoud et al. 1997; Phillips and Powley 2007). The prominent supply in the small intestines is from the meissner plexus, innervating primarily nutrient-sensors (Berthoud and Neuhuber 2000a, 2000b; Phillips and Powley 2007). The vagus also innervates the pancreas by hepatic and gastric branches, which provides innervation to both alpha and beta islet cells that release glucagon and insulin, respectively (Matthews and Clark 1987; Rodriguez-Diaz and Caicedo 2014). Spleen is considered a vital organ in the neuroimmune circuit of inflammatory reflex (Tracey 2002). The vagus innervates the spleen indirectly through the celiac ganglion (Bassi et al. 2020; Cailotto et al. 2012; Rosas-Ballina et al. 2008a, 2008b). Splenic nerve which innervates the spleen originates from the celiac ganglion (Bassi et al. 2020; Cailotto et al. 2012; Rosas-Ballina et al. 2008a, 2008b). The vast innervation of the vagus in the thoracic and abdominal organs offers an opportunity to control the physiology of the organs and implement neuromodulation therapy in disease processes.

### Fascicular structure of the vagus nerve trunk

The arrangement of fibers in the nerve plays an important role in neuromodulation therapies, as the activation or block of nerve fibers by electrical stimulation using an implanted electrode depends heavily on the distance between the electrode and the fibers: the shorter the distance, the smaller the stimulus intensity required to engage fibers (Grill and Mortimer 1997). Recently, preliminary studies in swine indicate that fascicles in the vagus are arranged in a “viscerotopic” manner: fascicles that contribute to an organ are clustered together inside the vagus trunk (Jayaprakash et al. 2022; Thompson et al. 2022). The fascicular structure of the vagus in swine is comparable to that of the human vagus—with some notable differences, including the smaller number of fascicles and greater morphological variability in the human nerve (Pelot et al. 2020). Recently, a preliminary report suggested that fascicles in the human cervical vagus split and merge every ~ 500 μm, another possible contributing factor to the variability (Upadhye et al. 2021). Such variability of the fascicular structure may contribute to the heterogeneity of patient responses and clinical responsiveness to cervical VNS. These anatomical complexities might also provide the anatomical basis for organ- and function-selective VNS (Jayaprakash et al. 2022; Thompson et al. 2022). Compared with humans and large animal models, the fascicular structure of the vagus in rodent models is relatively simple, with only 1 to 3 small fascicles; that property makes rodents a good model for investigating fiber recruitment in response to VNS without the confound of the fascicular organization.

### Anatomical and functional characteristics of vagus nerve fibers

In the vagus, there are different types of fibers, approximately 1-2 k in rodents and 50-60 k in humans in total (Nathalie Stakenborg et al. 2020). Although the fascicular organization of the cervical vagus varies widely from species to species, the fiber types remain relatively consistent. Based on morphological and corresponding electrophysiological differences, fibers are categorized into several types (Table 1) (Erlanger and Gasser 1924). The different axonal sizes, myelin, and possibly different ion channel populations, lead to distinct conduction velocities and electrical properties in response to external electrical stimuli. These morphological differences also determine that, during nerve stimulation, fiber recruitment follows an order according to fiber size: larger fibers are recruited first (at lower intensities), and smaller fibers are recruited last (at higher intensities) (Parker et al. 2017).

**Table 1:** Classification of vagal fiber types at the cervical level according to the Erlanger-Gasser scheme

Fiber type	Diameter (μm)	Act. threshold	Myelin	Conduction velocity (m/sec)	Function	Direction
**Aα**	13–22	Low	Yes	70–120	Motor innervation of laryngeal muscles	Eff.
**Aβ**	8–13	Low	Yes	40–70	Mechanosensation of laryngeal and pharyngeal mucosal surfaces	Aff.
**Aδ**	1–4	Interm.	Yes	5–15	Visceral mechanoreceptors (e.g. baroreceptors), pain, temperature	Aff.
**B**	1–3	Interm.	Yes	3–14	Preganglionic autonomic parasympathetic to viscera	Eff.
**C**	0.1–1	High	No	0.2–2	Visceral sensation of pain, temperature, inflammatory stimuli	Aff.
**C**	0.1–1	High	No	0.2–2	Postganglionic autonomic sympathetic to viscera	Eff.

The largest myelinated fibers of the vagus belong to the A-type group and comprise approximately 5–10% of all fibers in the cervical vagus. Within this group are several subgroups: Aα, Aβ, and Aδ fibers, with progressively decreasing sizes and conduction velocities (Table 1). Efferent Aα fibers innervate laryngeal muscles bilaterally, through superior and recurrent laryngeal nerve branches; they form a cluster in the cervical vagus, right next to the emergence of those branches (Settell et al. 2020). Afferent Aβ fibers convey sensory information from receptors of the muscle spindles or low threshold mucosal surface mechanoreceptors related to cough reflex [ref]. Afferent Aδ fibers take part in autonomic reflexes and convey sensory signals from low threshold mechanoreceptors in response to pain, crude touch, pressure, and temperature stimuli (Parker et al. 2017). When stimulated, Aδ fibers elicit a physiological response consisting of bradypnea or apnea, changes in systemic blood pressure, and inhibition of efferent vagal activity (Belvisi 2003). Aδ fibers also express TRPV1 (transient receptor potential cation channel subfamily V member 1), also known as the capsaicin receptor or vanilloid receptor 1 (Caterina et al. 1997; Nakagawa and Hiura 2006). The subgroup of Aδ-fibers plays a crucial role in respiratory regulation by transmitting important sensory information associated with the central inspiratory drive (Bozler and Burch 1951; Carr and Undem 2003; R. B. Chang et al. 2015; Hayashi et al. 1996; Paintal 1973).(Bozler and Burch 1951; Carr and Undem 2003; R. B. Chang et al. 2015; Hayashi et al. 1996; Paintal 1973).

Efferent B-fibers are myelinated, relatively smaller, and have a higher recruitment threshold than A-fibers (Ahmed et al. 2021a, 2021b; Ruffoli et al. 2011). B-fibers make up approximately 10–15% of the fibers in the cervical vagus and constitute preganglionic axons terminating into synapses in parasympathetic ganglia. Stimulation of these fibers can result in bradycardia, with an almost linear dose-response relationship (Y. C. Chang et al. 2020). The mechanism underlying heart rate reduction can be attributed to the acetylcholine released by vagal parasympathetic fibers (Loffelholz and Pappano 1985) that innervate the sinoatrial and the atrioventricular nodes, with negative chronotropic and dromotropic effects, respectively (Coote 2013; Garamendi-Ruiz and Gomez-Esteban 2019; Gordan et al. 2015). Studies have also shown that vagal B-fibers are also involved in the inflammatory reflex (IR), a neuro-immune reflex whose efferent arc starts with cholinergic neurons in the brainstem’s dorsal motor nucleus, that travel down the vagus nerve to activate acetylcholine-producing T-cells in the spleen (Andersson and Tracey 2012; Chavan and Tracey 2017; Pavlov et al. 2018). Preganglionic neurons of vagal efferent fibers also innervate the muscular and mucosal layers of the gut both in the lamina propria and in the muscularis externa and supply the intestine from the proximal duodenum to the distal part of the descending colon (Breit et al. 2018).

C-fibers are unmyelinated small fibers, characterized by the slowest conduction velocity. The C-fibers present in the cervical vagus account for the majority, approximately 65–80% of all fibers (Agostoni et al. 1957), with the properties of the slowest conduction velocity of 0.2–2 m/s and the highest recruitment threshold among fibers (Groves and Brown 2005). C-fibers are mainly consisting of postganglionic afferents, but may also represent postganglionic axons from the sympathetic chain that “hitch-hike” into the vagus (Kawagishi et al. 2008; Levy and Martin 1981). However, the sympathetic nervous system could also be activated through vagal-sympathetic reflexes (Ahmed et al. 2021a, 2021b; Ardell et al. 2015). Such vagal-sympathetic reflexes are mediated centrally by direct connections from the vagal sensory nucleus of the solitary tract (NTS) to the dorsal motor nucleus (DMN) of the vagus and sympathetic ganglia (Ardell et al. 2015; Armour and Ardell 2004; Sawchenko 1983). When vagal C-fibers are activated by stimuli, the direct physiological response includes nausea, coarse voice, coughing, changes in breathing patterns, and activation of the sympathetic autonomic system (Seki et al. 2014; Verlinden et al. 2016). There have been two phenotypes of capsaicin-sensitive nociceptive C-fibers (neural crest C-fibers and placodal C-fibers) that have been identified in the respiratory and esophagus tract (Kollarik et al. 2010; Undem et al. 2004). Unmyelinated C-fibers also express TRPV1 (Caterina et al. 1997; Nakagawa and Hiura 2006). In Paintal 1973, A.S. Paintal conducted a review of vagal reflex fibers identifying several vagal sensory receptors (Paintal 1973), including Type J receptor, aortic chemoreceptors, epicardial and pericardial vagal sensory receptors associated with C-fibers. In the gastrointestinal tract, there are many reports of unmyelinated gastric stretch receptors, gastric mucosal as chemoreceptors, and intestinal mucosal as mechanoreceptors. C-fiber might also involve in the inflammatory reflex, as evidence of cytokine-specific sensory neural signals were measured in the vagus nerve (Steinberg et al. 2016; Zanos et al. 2018).

## Strategies for precision vagus neuromodulation

**Fig. 1:**
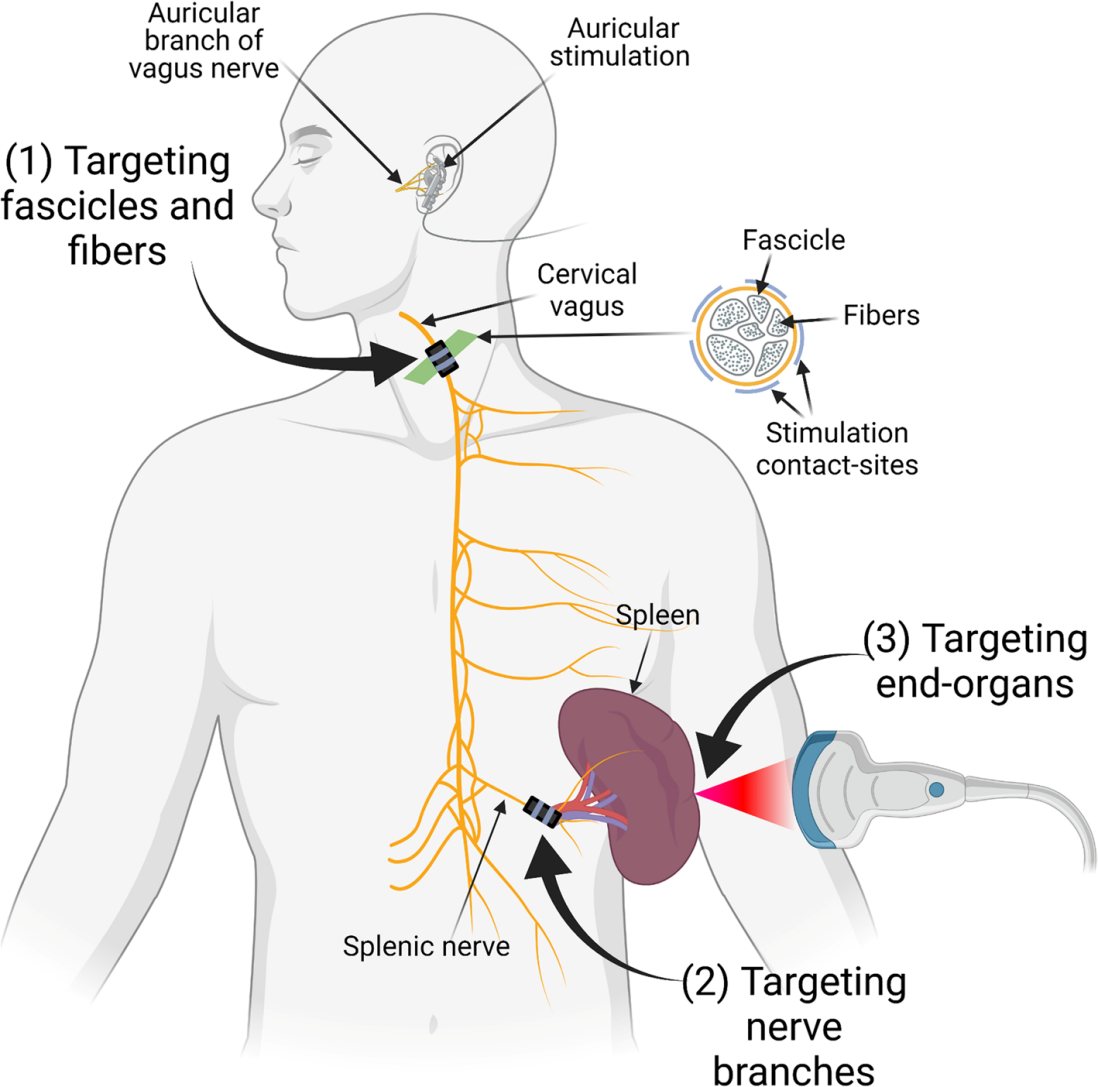
Illustration of strategies for precision vagus neuromodulation. The first strategy is to selectively stimulate fascicles and fibers of the vagus nerve at the cervical level. The second strategy is to stimulate the near-organ branch of the vagus. The third strategy is to directly stimulate the nerve endings at the end-organ

The extensive vagal innervation of visceral organs makes the vagus a target for neuromodulation therapies. The vagus is easily accessible at the cervical level and requires minor surgery to place an electrode for stimulation. However, the downside of the cervical vagus nerve is that it contains nerve fibers that innervate multiple organs, leading to unwanted adverse effects (Elinor Ben-Menachem 2001). Therefore, precise stimulation of the vagus nerve is needed to selectively affect certain fiber populations and organs and avoid others. Herein, we describe 3 potential strategies for precision VNS (Fig. 1).

### Targeting the vagus trunk at the cervical level

#### Fascicle-specific VNS

The first strategy for precision vagus neuromodulation is fascicular selectivity at the level of the cervical vagus nerve (Fig. 1). Vagal fibers at the cervical level are organized in a fascicular pattern that could be leveraged during VNS (Settell et al. 2020). One way of achieving fascicular selectivity is by placing a multi-contact cuff electrode around the cervical vagus nerve (cVN). This type of electrode permits targeting a specific fascicle using a combination of two or three contact points through current steering. Fascicular selectivity would allow to maximize the desired effects and minimize the unwanted adverse effects of cVN. This approach was used in a rodent model where the investigators have selectively reduced blood pressure without affecting heart rate and breathing rate by using multi-contact electrodes on cVN (Gierthmuehlen and Plachta 2016). Their results indicate that the baroreceptors fibers in cVN responsible for controlling blood pressure can be selectively activated using a multi-contact electrode (Gierthmuehlen et al. 2016). Aristovich et al. has also used this method in a large animal model (Aristovich et al. 2021), sheep, where they spatially stimulated the cervical vagus nerve and demonstrated that a fascicular stimulation could affect the breathing rate without affecting the heart rate. By changing the selected electrode contact points in a multi-contact electrode and targeting different fascicle, they were able to affect heart rate without any change in breathing rate (Aristovich et al. 2021). This study is critical in translational research because the size and fascicular organization of the large animals are close to humans (Settell et al. 2020; Nathalie Stakenborg et al. 2020), The findings also imply that most of the fibers with the same functionality likely present in the same fascicle at the cervical level of the vagus nerve, which gives rise to the potential to achieve precision VNS for specific organ through spatial selectivity.

A second method for fascicular selectivity is the use of an intraneural electrode that penetrates the vagus nerve and only stimulates those fascicles closest to selected electrode contacts. A study in mice showed the feasibility of using a needle electrode to effectively stimulate the vagus nerve percutaneously (Huffman et al. 2019). Another study in rats and swine demonstrated the use of penetrating electrodes to record intraneural vagal activity (Jiman et al. 2020; Vallone et al. 2021); the extent to which such an electrode can be used for selective stimulation is unknown. A third potential way for attaining fascicular selectivity is with a flat electrode instead of the traditional circumferential electrodes. This type of electrode compresses and “expands” nerve tissue along its surface area, thereby separating functionally distinct nerve fibers enough to be selectively stimulated through different electrode contacts (Bucksot et al. 2019). However, neither of these methods has been tested in the context of fascicle-specific VNS.

#### Fiber-specific VNS

The vagus conveys sensory and motor information through different populations of afferent and efferent fibers, mediating different functions (Table 1). Almost all these fibers go through the cervical vagus. That means that many vagal functions are in principle accessible to precision neuromodulation through a cervical vagus electrode. In the following section, we discuss approaches for direction-specific or fiber type-specific precision VNS.

#### Direction-specific VNS

Anodal block is a well-documented technique that has been used to suppress the large myelinated fiber associated response, such as laryngeal muscle contraction, or bias the afferent/efferent pathway of VN, thus eliciting preferential vagal effects (U. Ahmed et al. 2020; Vuckovic et al. 2008). Through hyperpolarization of the nerve fiber near an anode, the action potential is biased to propagate in the opposite direction. As the myelinated fibers are more sensitive to hyperpolarizing current, the threshold for blocking such fiber is much less than smaller fibers, making it an option for targeting afferent or efferent pathway, through cathode-cephalad or cathode-caudad stimulation polarity configurations, respectively (U. Ahmed et al. 2020). However, controlling the proper stimulus intensity is critical for anodal block, which makes it difficult to accomplish perfect directional activation as the unpredictable distribution of nerve fiber within the cervical VN, as well the insensitivity of unmyelinated fiber.

#### Fiber type-specific VNS

Waveform manipulation, together with anodal block, is the other technique used to improve differential vagal fiber activation. Several general waveforms have been used, including slowly rising (or triangular) pulses (Hennings et al. 2005a, 2005b; Jones et al. 1995; Vuckovic et al. 2008), pre-pulse (Vuckovic et al. 2008), and quasi-trapezoidal (QT) (or exponential falling) (Y.-C. Chang et al. 2021; Tosato et al. 2007; Vuckovic et al. 2008). The mechanism for a slowly-rising pulse can be attributed to the different spatial distribution of the ion channels for large and small nerve fibers, as the distant nodes of Ranvier of larger fiber are hyperpolarized more than a distant node of smaller fibers; however, the adequate pulse duration and the slope is important to achieve good selectivity (Hennings et al. 2005a, 2005b). For depolarizing prepulse, the nerve fiber is first conditioned with subthreshold current, resulting in inactivation of the voltage-dependent sodium channel and increase of excitation threshold (Grill and Mortimer 1995, 1997). Such a type of pulse has limitation originating from the unique waveform, as the prolonged phase leads to high charge injection and limits the stimulation frequency. The QT pulse is designed to depolarize the nerve under cathode and simultaneous selective blockade under anode (Fang and Mortimer 1991), and has been validated theoretically and experimentally in functional implanted devices (Bhadra and Mortimer 2005). QT has first been shown with a promising effect on VNS to prevent laryngeal spasms (Tosato et al. 2007). In a waveform comparison study using a swine model, with evoked compound action potential as quantitative measurements, a slow rising pulse can achieve a 60% of reduction in Aβ activity, whereas depolarizing prepulse achieved up to a 90%. The QT completely prevented Aβ activation in two out of five animals, and reduce 60–90% in the three. QT has also been found to selectively activate B-fiber and result in corresponding bradycardia without much laryngeal muscle contraction in rodent models (Y.-C. Chang et al. 2021).

Intermittent burst of square pulses, or so-called chopped pulse with frequency around 10–50 Hz, has been used to achieve selective stimulation of the vagus. The underlying concept is using the earlier pulses in the burst to condition the sodium channels into an inactivation state, primarily in large fiber, and allowing smaller fibers to be preferentially excited by the later pulses. In a rat model, Qing et al. have compared the chopped pulse with normal rectangular pulses at 50% charge level required to elicit maximum C-fiber response and found the chopped pulses were able to maintain similar C-fibers response while reducing A-fiber by 11% (Qing et al. 2015), though the C-fiber response derived from compound action potential has faster conduction velocity than most of the other studies. In the dog model, Yoo et al. has leveraged a similar method and demonstrated comparable HR modulation while reducing laryngeal side effects, indicated by the amplitude of electromyography signal extracted from corresponding muscles, compared with regular VNS. Although the authors claim that chopped pulses displayed comparable efficacy, in terms of change of heart rate, to regular non-selective VNS, the actual efficacy for proposed method, and how it translational to clinic, might require further investigation, as this finding does not apply to all stimulation intensities, especially those above the bradycardia threshold (Yoo et al., 2016).

High-frequency stimulation, also called kilohertz electrical stimulation (KES), uses frequently alternating (> 1 kHz) rectangular or sinusoidal current to induce reversible neuromodulation. The majority of studies investigated inhibitory effects that associate with neural conduction block (Neudorfer et al. 2021), and most early studies tested with somatic nerve while monitoring the large efferent fiber response and related muscle force (Kilgore and Bhadra 2014). The actual mechanism behind the KES is still under debate, but is most likely related to the dynamic of ion channels, especially sodium channels (Yoshida et al. 2018; Zhao et al. 2018). Depending on the dose of a single pulse, the KES response can be mainly categorized into inhibitory and excitatory effects, triggered by supra- and sub-threshold intensities, respectively. When applying the neural level supra-threshold stimulation at frequencies above the neuron’s normal physiological range (0.05 Hz–500 Hz), the nerve will demonstrate conduction block and associated phenomena, including onset response, desynchronization, and spike-rate adaptation, as consecutive stimuli occur within absolute refractory period lead to random open-close kinetics of voltage-gated sodium channels that ultimately bias the membrane potential towards the more depolarized or hyperpolarized state (Clay and DeFelice 1983; Sigworth 1980). In contract, when delivering stimuli at the subthreshold level, the nerve most likely generates action potentials via integrative properties, namely facilitation, which is similar to temporal summation at the synaptic level (Boulet et al. 2016). The net effect of KES highly depends on the frequency, amplitude, and overall duration of the applied stimulus. The KES resulted in block effect is not limited to larger fibers but all fiber types, and responses are recoverable with proper stimulation dose range and recovery time (Pelot and Grill 2020). In VN, recently, KES nerve block has been applied to enhance the anti-inflammatory effect (Kilgore and Bhadra 2014), satiety, and appetite (Apovian et al. 2017; Johannessen et al. 2017). On the other hand, KES has been also reported to provide excitatory effect, especially in the auditory nerve (Heffer et al. 2010) and retinal ganglion cells (Guo et al. 2019), which allows generation of action potentials via temporal summation of subthreshold stimuli (Neudorfer et al. 2021). Recently it was suggested that KES used in a VN neuromodulation therapy of obesity (Enteromedies vBloc) may exert its actions via fiber activation, rather than blocking (Johannessen et al. 2017; Pelot et al. 2017). Combined with the excitatory and inhibitory capability, one recent study has shown that the intermittent KES can be leveraged to achieve specific C-fiber selective activation in rodent VN through the frequency and intensity interaction (Y.-C. Chang et al. 2021).

Other than directly leveraging the electrophysiological property differences across fibers for selective VNS, the neural fulcrum is defined as the operating point, for common stimulation parameter variations, that can reach a dynamic equilibrium of vagal control of cardiac function (Ardell et al. 2017), as both afferent and efferent VN fiber demonstrate opposite effects on HR modulation (decrease of central parasympathetic drive vs. increase of cardiac parasympathetic drive) (Ardell et al. 2015). The results show that at low intensities and higher frequency VNS, HR tends to increase due to afferent modulation of parasympathetic central drive. As intensity further increase, passing the ‘neural fulcrum’ equilibrium point, HR tends to reduce during the VNS. Another recent study has demonstrated the effect of intensity on fiber activation by recording evoked compound action potentials, and physiological responses by recording systemic arterial pressure (SAP), heart rate (HR), and breathing rate (BR) (Ahmed et al. 2021a, 2021b). Their results indicate that using the stimulation parameters of square waveforms and 30 Hz frequency, at low intensity, A-fibers are activated along with the decrease in BR (bradypnea) and increase in SAP. At intermediate intensity, B-fibers are also activated along with a decrease in BR (bradypnea), HR, and SAP. At high intensity, C-fibers are also activated along with a greater drop in BR (apnea), HR, and SAP. Interestingly, the stimulation intensity needed to elicit a similar physiological response changes significantly during anesthetized vs. awake states (Ahmed et al. 2021a, 2021b). Overall, this idea can also be applied to optimizing the therapy by maximizing desired effects versus the others.

### Targeting near-organ vagal branches

The second strategy for precision vagus neuromodulation is to target branches of the vagus nerve near the end-organs (Fig. 1). The vagus innervates most visceral organs, including the heart, lungs, liver, spleen, and gastrointestinal tract. Therefore, only the concerned branch of the vagus nerve can be targeted without disturbing the physiology of other organs (Falvey et al. 2022). This approach has been used before in autonomic neuromodulation, in stellate ganglion stimulation for modulation of atrio-ventricular conduction (Zipes et al. 1974), and later applied to the vagal gastric branch for modulating gallbladder responses (Furukawa and Okada 1992). Spleen is another major organ with vagal involvement, in particular its cholinergic anti-inflammatory pathway (Huston et al. 2006). Splenic nerve stimulation can be used to suppress inflammation in autoimmune or inflammatory diseases, to yield similar results as cervical VNS. A recent study has utilized splenic nerve stimulation during acute inflammation in an anesthetized pig model (Donega et al. 2021). Their results indicate that splenic nerve stimulation can enhance the anti-inflammatory response during acute inflammation without changing other physiological functions such as heart rate and breathing rate (Donega et al. 2021). Additionally, the cytokine suppression with splenic nerve stimulation was similar to traditional cervical vagus nerve stimulation. Another study has demonstrated the same anti-inflammatory effects of splenic nerve stimulation in a low-dose endotoxemia chronic pig model (Sokal et al. 2021). They have shown that splenic nerve stimulation reduces pro-inflammatory cytokine tumor necrosis factor-a and pro-inflammatory eicosanoids, including prostaglandins. These studies suggest that targeting vagal innervation at the branch level could potentially maximize desired effects and minimize unwanted adverse effects. However, further studies are needed to test the anti-inflammatory effects of splenic nerve stimulation in chronic inflammatory diseases before testing this method in humans.

The abdominal vagus trunk is a nerve target for moderate to severe obesity. In the ReCharge clinical trial, the Maestro Rechargeable System consists of two leads placed around the anterior and posterior vagal using standard minimally invasive laparoscopic surgical techniques (Ikramuddin et al. 2014). Through delivering low energy, high frequency, intermittent, electrical pulses to the intra-abdominal vagal trunks for a predetermined number of hours each day, the participants show significant weight loss with more tolerable side effects. The underlying mechanism was first associated with intermittent vagal blockage which is believed to reduce the sensations of hunger (Apovian et al. 2017); however, the most recent study shows that the system might be modulated through continuous nerve activation rather than blockage (Pelot et al. 2017). The major obstacle of this approach is that not every organ innervated by the vagus can be easily targeted, such as the heart and lungs. Placement of the electrode on the cardiac or bronchial branches would require major surgery, and the benefits may not outweigh the risks of the surgery. Therefore, additional preclinical research is necessary to assess the feasibility of targeting vagal innervation at the branch level, such as the hepatic, cardiac, and bronchial branches of the vagus nerve.

### Targeting vagal terminals at the end-organs

Perhaps the most intuitive strategy to regulate specific organs through VNS is to directly stimulate the nerve terminals after they enter the end-organ (Fig. 1). The possibility to target those relatively small or deeper organs is through other physical modalities, such as focused ultrasound stimulation (FUS), with its ability to penetrate soft tissue while allowing decent focus. An example of FUS at the end-organs, such as the spleen in case of inflammatory diseases or pulmonary arterial hypertension (Ahmed et al. 2021a, 2021b; Zachs et al. 2019) An example of FUS at the end-organs, such as the spleen in case of inflammatory diseases or pulmonary arterial hypertension (Ahmed et al. 2021a, 2021b; Zachs et al. 2019). The spleen is a principal organ involved in the cholinergic anti-inflammatory pathway related to the vagus nerve (Huston et al. 2006; Rosas-Ballina et al. 2011). In acute rodent endotoxemia models, FUS at the spleen has been shown to reduce cytokine response to endotoxin to a level similar to implant-based cervical VNS (Cotero et al. 2019). FUS of the spleen has also been shown to reduce severity in certain chronic diseases where inflammation plays a crucial role in disease progression. A study in a mice model of rheumatoid arthritis has demonstrated significant improvement in the disease severity by utilizing daily FUS at the spleen for 1 week (Zachs et al. 2019). They have shown a substantial reduction in the joint swelling in the FUS group compared to controls. One of the possible mechanisms of action behind this finding was investigated by looking at the gene expression profile of lymphocytes in the spleen using single-cell RNA sequencing. The pro-inflammatory gene expression was significantly reduced in the animals that received FUS. Thus, confirming the anti-inflammatory effects of FUS at the spleen. Another study in a rat model of pulmonary hypertension has shown a significant reduction in right ventricular systolic pressure in the animals that received daily FUS at the spleen for 2 weeks (Ahmed et al. 2021a, 2021b). The results also indicate that FUS at the spleen does not affect heart rate or systemic arterial pressure. The change in heart rate is particularly important in patients with PAH, a slight drop in heart rate can worsen the cardiac output, resulting in further enhancement of the signs and symptoms of PAH. These results indicate that targeting vagal fibers at the end-organs attains the required effects and reduces the side effects observed by affecting other organs innervated by the vagus nerve. Another study has shown that FUS at the liver improves metabolic functions related to obesity (Huerta et al. 2021). They have demonstrated that FUS at the liver reduces body weight, circulating lipids, hepatic inflammatory cytokine levels, and hepatic leukocytes infiltration (Huerta et al. 2021). A more recent study has implemented the same FUS at the porta hepatis in diabetes mellitus type-2 (DM) in 3 animal species (mice, rats, and swine) (Cotero et al. 2022). They found out that FUS reduces blood glucose levels both acutely and chronically. In acute experiments, their results indicate that a single 3-minutes of FUS can lower blood glucose and insulin level, bringing the glucose homeostasis close to the healthy state. They observed similar results in chronic experiments when they stimulated daily for 8 weeks. The mechanism behind the reduction in glucose levels is the hepatoportal glucose-sensing phenomenon in which nerve signals report hepatic artery-portal vein glucose gradients during feeding or fasting, which results in neuronal modulation of the metabolic system. In addition, they demonstrated that mechanosensitive ion channels at the portahepatis are responsible for this effect (Cotero et al. 2022). Collectively, these studies indicate that FUS could be used as a precise and non-invasive way of targeting vagal fibers at the level of the end-organ.

The stimulation pulse frequencies used in the previous studies are consistent between 1 MHz to 1.1 MHz (Cotero et al. 2019; Huerta et al. 2021; Zachs et al. 2019). However, Zach. et al. has tested multiple frequencies ranging from 220 KHz – 1 MHz (Zachs et al. 2019). Interestingly, they found that stimulation frequency of 1 MHz produces the most beneficial results, similar to other studies. In the same studies, the pulse repetition period ranges from 0.5–200 msec and burst period of 150 pulses. The range for the stimulation duration used was 2–20 min; however, 2 min of daily stimulation was sufficient to produce significantly favorable results, regardless of the acute or chronic disease models.

The mechanism underlying FUS modulation of the nervous system remains unclear. However, several studies have shown that FUS can activate or modulate peripheral nerves (Downs et al. 2018; Kim et al. 2012; Lele 1963), possibly through a mechanical or thermal effect that actuates or modulates voltage-gated ion channels or mechanosensitive ion channels on neural tissue membranes, or through a cavitational effect resulting in direct ionic flux (W. J. Tyler et al. 2008; Wright et al. 2017). FUS has also been shown to activate skin receptors in humans and other excitable cell types or induce cell membrane porosity (Cotero et al. 2022; Kubanek et al. 2018; Legon et al. 2012). The key clinical advantage of FUS is the non-invasive precise targeting of the nerves within the end-organ, making it a unique tool in neuromodulation therapy.

### Noninvasive vagus nerve stimulation

The auricular branch of the vagus is another target of interest, especially for applications related to vagal afferents terminating in the nucleus of the solitary tract (NTS), such as epilepsy and neuroinflammatory related diseases, as it carries somatosensory signals from the ear with the same projection at NTS. With its superficial nerve ending near the ear (tragus and auricle), the auricular vagus nerve stimulation (aVNS) can be delivered percutaneously or transcutaneously, offering a method to modulate neural activity on the vagus nerve with the potential for a more favorable safety profile. Given aVNS can be implemented with minimally invasive approaches and has the potential to modulate vagal activity, there have been many early-stage clinical trials investigating a diverse range of potential therapeutic indications, including heart failure, epilepsy, depression, pre-diabetes, Parkinson’s, and rheumatoid arthritis, and several aVNS devices were already developed for various applications (Verma et al. 2021).

Cervical, non-invasive VNS (nVNS) devices, such as GammaCore (NJ, USA), are designed primarily to stimulate myelinated sensory afferent vagus nerve fibers as they ascend through the neck in the carotid sheath, using a battery-powered external electrical stimulator. This device has been approved and is being prescribed in several countries mainly for the treatment of primary headache and is CE marked in the EU for the treatment of primary headache, epilepsy, bronchoconstriction, anxiety, depression, and gastric motility disorders (Mwamburi et al. 2017; Robinson et al. 2020). More recently, this device was also tested in a short-term proof-of-concept study for patients with treatment-refractory gastroparesis, as an alternative for implantable gastric electric stimulation devices (Paulon et al. 2017).

Although both aVNS and nVNS provide non-invasive therapies which significantly eliminate the safety risks, anatomically, their main primary target is the vagal afferent pathway which has no direct association with their current implications. Many questions remain regarding the efficacy of this therapy, as there is currently no firm evidence regarding underlying neurophysiological mechanisms for such neuromodulation (Yap et al. 2020). The potential use of and mechanisms for noninvasive vagus therapies in a precision neuromodulation context is still under investigation.

### Technologies needed for clinical implementation of precision neuromodulation

Today, there are sufficient technologies to support these three strategies for precision vagus neuromodulation. For targeting the vagus at the cervical level, fascicle-selective stimulation can be delivered through the multi-contact electrode, and fiber-selective stimulation can be delivered by using specific-stimulation waveforms and parameters. The major advantage of cervical vagus stimulation is the ease of accessibility and requires a relatively minor surgical procedure for electrode implantation. The disadvantages of this strategy are the lack of clinical trials that have implemented the latest multi-contact electrodes or selective stimulation parameters, as all the previous studies were conducted in pre-clinical animal models. For targeting the vagus at the branch level, a relatively simple bipolar or tripolar electrode with conventional stimulation parameters would be sufficient to get beneficial results. However, the benefits of the desired results need to outweigh the risks of the surgical electrode implantation at the branch level, especially for deeply located organs. For targeting vagal fibers end-organs, the biggest attraction of this strategy is the non-invasive neuromodulation with the use of ultrasound. This can provide precise stimulation of the nerve endings at the end-organs and can significantly avoid unwanted adverse effects. Another advantage is the straightforward translation from pre-clinical to clinical studies, as the ultrasound is readily available in the hospitals, and will also make it easy for the clinicians to recruit patients. The disadvantage of this strategy is the difficult access to the deeply located organs which can limit the use of end-organ stimulation. Additionally, an ultrasound machine is needed which is operated by a healthcare worker. Simple and compact ultrasound devices are needed that can deliver daily ultrasound stimulation without the need for a trained operator.

## Translational applications of precision vagus neuromodulation

### Neurological disorders

In most of neurological disorders in which VNS is used (Table 2), the target is the afferent fibers in the cervical vagus with their direct projections in different brain areas and subsequent neuronal actions, or afferent and efferent fibers involved in inflammatory reflexes with subsequent suppression of neuroinflammation.

**Table 2:** Evidence and feasibility of therapeutic strategies for precision vagus neuromodulation in different disorders

Disorder	Targeted organ/ process	Targeted anatomical element of vagus	Evidence for/Feasibility of Targeting Strategies		
			Cervical VNS	Near-organ (at-branch) stimulation	At-organ ultrasound stimulation
Epilepsy	Brain cortical excitability	Afferent vagus fibers	Feasible (E. Ben-Menachem et al., 1994; zHandforth et al., 1998)	Unclear/ Not feasible	Unclear/ Not feasible
Depression	Brain monoamine system	Afferent vagus fibers	Feasible (Bajbouj et al., 2010; Sackeim et al., 2001)	Unclear/ Not feasible	Unclear/ Not feasible
Headaches (Migraine and Cluster)	Neurovascular	Afferent/efferent vagus fibers	Feasible (Barbanti et al., 2015; Silberstein et al., 2016)	Unclear/ Not feasible	Unclear/ Not feasible
Stroke Rehabilitation	Cortical plasticity	Afferent vagus fibers	Feasible (Dawson et al., 2021; Dawson et al., 2016; Engineer et al., 2019)	Unclear/ Not feasible	Unclear/ Not feasible
Multiple Sclerosis	Brain and spinal cord, inflammation	Afferent/efferent vagus fibers	Feasible (Marrosu et al., 2007)	Unclear/ Not feasible	Unclear/ Not feasible
Tinnitus	Cochlea	Afferent vagus fibers	Feasible (R. Tyler et al., 2017; Wichova et al., 2018)	Unclear/ Not feasible	Unclear/ Not feasible
Heart Failure	Heart	Efferent autonomic vagus fibers (B-type, C-type), cardiac n.	Feasible (De Ferrari et al., 2011; Gold et al., 2016)	Feasible	Feasible
Arrhythmias	Heart	Efferent autonomic vagus fibers (B-type, C-type), cardiac n.	Feasible (Ando et al., 2005; Zhang & Mazgalev, 2011)	Feasible	Unclear/ Not feasible
Hypertension	Carotid body	Afferent vagus fibers (Αδ-type and C-type), aortic depressor n.	Feasible (Annoni et al., 2019)	Feasible (Gierthmuehlen & Plachta, 2016)	Unclear/ Not feasible
Pulmonary Hypertension	Lung vessels, right ventricle, inflammation	Efferent vagus fibers, bronchial n., splenic n.	Feasible (Ntiloudi et al., 2019; Yoshida et al., 2018)	Feasible	Feasible (Umair Ahmed et al., 2021a, 2021b)
COVID-19 ARDS	Lungs, inflammation	Afferent/efferent vagus fibers, bronchial n., splenic n.	Feasible (Fudim et al., 2020; Li, Qi, Li, Deng, & Wang, 2021; Mastitskaya, Thompson, & Holder, 2021)	Unclear/ Not feasible	Unclear/ Not feasible
Rheumatoid Arthritis	Spleen, inflammation	Afferent/efferent vagus fibers, splenic nerve	Feasible (Koopman et al., 2016)	Feasible	Feasible (Zachs et al., 2019)
Gastroparesis	Stomach	Afferent/efferent vagus fibers, subdiaphragmatic vagus	Feasible	Feasible (Malbert, Mathis, & Laplace, 1995)	Feasible
Inflammatory Bowel Disease	GI tract, inflammation	Afferent/efferent vagus fibers, subdiaphragmatic vagus	Feasible (Bonaz, Sinniger, Hoffmann, et al., 2016)	Feasible (Caravaca, Levine, Drake, Eberhardson, & Olofsson, 2021; S. C. Payne et al., 2019)	Feasible (Nunes et al., 2019)
Postop. Intestinal Obstruction	GI tract motility	Afferent/efferent vagus fibers, subdiaphragmatic vagus	Feasible (N. Stakenborg et al., 2017)	Feasible (N. Stakenborg et al., 2017)	Feasible
Diabetes	Liver and pancreas	Afferent/efferent vagus fibers, hepatic and pancreatic n.	Feasible (Fontaine et al., 2021; Yin, Ji, Gharibani, & Chen, 2019)	Feasible(Chen, Pasricha, Yin, Lin, & Chen, 2010; Jon J. Waataja, 2021; Lee & Miller, 1985; Sophie C. Payne et al., 2022; S. C. Payne et al., 2020)	Feasible (Cotero et al., 2022)
Fibromyalgia	Inflammation	Afferent/efferent vagus fibers	Feasible (Lange et al., 2011)	Unclear/ Not feasible	Unclear/ Not feasible
Lupus	Inflammation	Afferent/efferent vagus fibers	Feasible (Mathis, Stauss, Pham, Kim, & Kulp, 2018)	Feasible	Feasible
Obesity	Liver (porta hepatis)	Afferent vagus fibers, subdiaphragmatic vagus, hepatic n.	Feasible (Bodenlos et al., 2007; Val-Laillet, Biraben, Randuineau, & Malbert, 2010; Yao et al., 2018)	Feasible (Ikramuddin et al., 2014)	Feasible (Huerta et al., 2021)

Almost every form of epilepsy is associated with brain neuroinflammation (Rana and Musto 2018; Vezzani et al. 2019; Vezzani et al. 2011), with a demonstrated positive correlation between neuroinflammatory markers and frequency of seizures (Boer et al. 2006; Pracucci et al. 2021). Whether neuroinflammation is the initiating cause of epilepsy or it develops when the disease progresses is under debate (Pracucci et al. 2021).

VNS is known to suppress systemic inflammation through a phenomenon known as inflammatory reflex (Tracey 2002). Efferent fibers in the vagus activates T-cells in the spleen, which suppresses the cytokine release from macrophages resulting in inducing systemic anti-inflammatory response (Bonaz et al. 2016a, 2016b). Afferent fibers in the vagus are also known to activate the same pathway through a central reflex mechanism (Bonaz et al. 2017). In addition to suppressing systemic inflammation, it has been shown that VNS also suppress neuroinflammation in the brain (Namgung et al. 2022). Currently, VNS is an FDA approved therapy for treatment resistant epilepsy (zHandforth et al. 1998). The therapeutic mechanism is still not well understood. Stimulation parameters used in clinical settings likely only stimulate low threshold, afferent A-fibers. Efferent B-fibers are likely minimally stimulated with the presently approved intensity range. Whether afferent and/or efferent VNS is more effective at suppressing inflammation and neuroinflammation is unknown; given that vagus fibers in the cervical region are involved in both afferent and efferent arcs of inflammatory reflexes, selective approaches will be needed to resolve this question. It has been shown that anti-inflammatory drugs may have a beneficial effect in epilepsy (Dey et al. 2016; Yamanaka et al. 2021). To our knowledge, there are no studies that have investigated the role of VNS in the context of neuroinflammation and epilepsy. Suppressing neuroinflammation associated with epilepsy via targeted VNS may be a strategy to maximize the anti-epileptic efficacy of VNS. Studying the effects of VNS on neuroinflammatory markers in models of epilepsy and patients with epilepsy may reveal novel VNS parameters and targets with stronger anti-epileptic activity. Similarly, stroke is also known to cause neuroinflammation in the brain (Amruta et al. 2020; Jayaraj et al. 2019), and the same opportunity of selective vagus nerve stimulation may be relevant in stroke.

### Cardiovascular disorders

Cardiovascular disorders such as heart failure, arrythmias, hypertension, and pulmonary hypertension are few of the examples in which two of the described strategies can be implemented, cervical and near-organ VNS. Cervical VNS is commonly utilized in the pre-clinical and clinical studies; however, due to the adverse effects on other organs, stimulation at the cardiac branch of the vagus would only affect the cardiac fibers.

There is an expanding body of clinical evidence for the use of cervical VNS in HF with reduced ejection fraction (HFrEF). The three largest clinical trials of cervical VNS (i.e. NECTAR-HF, INOVATE-HF, ANTHEM-HF) in patients with HFrEF on top of optimal medical treatment yielded in mixed results (Gold et al. 2016; Premchand et al. 2014; Zannad et al. 2015). These discrepancies could be due to major differences in the stimulation parameters, targets and systems. Also, off-target effects of cervical VNS including hoarseness, dysphonia, cough, GI discomfort, neck twitch and shortness of breath, often hindered VNS dose up-titration to recommended therapeutic levels. The pivotal ANTHEM-HFrEF is based on the concept of neural fulcrum (Ardell et al. 2017) is ongoing and will provide insights into the feasibility of cervical VNS for HFrEF. An alternative approach to cervical VNS for HF, would be the placement of the electrode on the cardiac branch of the vagus nerve. Theoretically, this approach could minimize the off-target effects and allow appropriate dose titration. However, it would require an open-heart surgery and expose patients to a high risk of postoperative complications, which probably outweighs the potential benefits. It would be of great interest a pilot study with cardiac branch stimulation in HFrEF patients that undergone an open-heart surgery for a different reason (coronary artery by-pass graft or valve surgery).

A noninvasive approach for HF patients is the stimulation of the auricular branch of the vagus nerve. LLTS has been mainly tested in patients with HF with preserved ejection fraction (HFpEF). Recently, two randomized and double-blind clinical trials showed that LLTS reduces TNF-a, improves left ventricular strain and quality of life in this population (Stavrakis et al. 2022). As treatments for HFpEF are lacking, LLTS promising preliminary results warrants further study in this disease.

In a plethora of animal studies, low-level cervical vagus nerve stimulation, namely stimulation at an intensity 10–50% of the threshold of heart rate reduction, suppressed atrial fibrillation (AF) inducibility and duration. These findings were also consistent with an LLTS study in canines. Notably, LLTS reduces pacing-induced AF burden and inflammatory cytokines in humans with paroxysmal AF at the electrophysiology lab and also in a pilot randomized sham-controlled trial with chronic low-level tragus stimulation in patients with paroxysmal AF, LLTS resulted in 85% lower AF burden at 6 months along with 23% lower TNF-α levels compared to the sham group.

### Gastrointestinal disorders

The vagus nerve (VN) innervates all the major gastrointestinal (GI) organs in the abdominal cavity. Afferent and efferent nerves within the branches of the VN enter and leave the GI organs. Selective stimulation of the VN innervating GI organs can be accomplished by all three strategies, either at the cervical level, at near-organ branch level, and on-organ through ultrasound stimulation. Here, we discuss studies related to GI disorders that use or could benefit from using prevision vagus neuromodulation.

Recently, VNS was proposed to treat inflammatory bowel disease, based on its ability to suppress inflammation through inflammatory reflexes. Several animal studies have demonstrated the anti-inflammatory effects of cervical VNS on animal models of IBD (Bonaz et al. 2016a, 2016b; D'Haens et al. 2019; Jin et al. 2017; Kibleur et al. 2018; Meregnani et al. 2011; Sun et al. 2013). Despite numerous clinical and preclinical investigations, there is lack of studies that have utilized fascicle- or fiber- specific stimulation that can even further potentiate the therapeutic efficacy. Payne et al. utilized near-organ VNS by targeting the abdominal vagus nerve in the intestinal inflammation animal model, and they found the similar therapeutic anti-inflammatory results as cervical VNS (S. C. Payne et al. 2019). However, targeting abdominal vagus did not produce any adverse effects related to cardiovascular and respiratory systems. Finally, Nunes et al. showed the improvement in colitis animal model by utilizing ultrasound stimulation at the abdomen (Nunes et al. 2019). These studies indicate that in the gastrointestinal diseases all of the three strategies of precision vagus neuromodulation can be implemented.

Postoperative ileus (POI) is a common disorder in patients undergoing abdominal surgery. The pathogenesis of POI is thought to be due to cytokine release during and after the surgery. TNF-α released from inflammatory muscular lamina propria and activated permanent macrophages (de Jonge et al. 2003). In the same manner that VNS can reduce the inflammatory response in IBD it can decrease the inflammatory response to intestinal surgery (The et al. 2007). This anti-inflammatory effect is mediated by macrophage activation and cytokine reduction which is driven by inflammatory reflexes (Munyaka et al. 2014). Stakenborg et al. showed in preclinical models that preoperative VNS could reduce inflammation caused by surgery and prevent POI and confirmed that the anti-inflammatory effect was caused by ACh acting on α7nAChR located in macrophages (N. Stakenborg et al. 2019). In preliminary studies Stakenborg et al. stimulated abdominal VN in POI mice and found that TNF-α levels decreased, and that intestinal transport significantly improved (N. Stakenborg et al. 2017). In human trials, Hong et al. tested whether noninvasive auricular electrical (EA) percutaneous vagus stimulation affects inflammation in POI models (Hong et al. 2019). The results showed that EA activated nucleus of the tractus solitarius and DMV, decreased the expression of intestinal cytokines, and reduced the recruitment of white blood cells to the intestinal segment of the operation, which improved the gastrointestinal transport after an operation.

Obesity, defined as body mass index (BMI) of 30 kg/m^2^ or more, is a rapidly growing public health and a potential indication for neuromodulation. Afferent communication between the gut and the brain are important in satiety, appetite, and hunger (Brookes et al. 2013; Kentish and Page 2015) and the vagus nerve is important in transmitting these signals from the gastrointestinal tract to the brain. Studies have demonstrated that many of the hormones secreted from enteroendocrine cells in the gut, signal through the vagus leading to either hunger or satiety (Cork 2018). Vagal mechanosensitive afferent fibers also give rise to intraganglionic laminar endings and intramuscular arrays within the stomach wall that signal both tension and stretch.. Thus, the mechanism of action for the treatment of obesity through VNS appears most likely to induce satiety by modulation of the gut–brain neural axis, gut peptide hormone release, and gastric motor activity. A retrospective assessment of patients with epilepsy and depression implanted for 2 years with a VNS device showed that stimulation therapy resulted in 5–10% reduction in total body weight (Burneo et al. 2002; Pardo et al. 2007). Animal studies have shown that VNS with low frequency (0.1–1 Hz) and long pulse width, results in a decrease in food intake and decrease in weight gain for normal and diet-induced obese rats (Bugajski et al. 2007; Laskiewicz et al. 2004; Laskiewicz et al. 2003). VBLOC therapy, is a vagus nerve selective therapy that involves surgical placement of cuff-like electrodes around the anterior and posterior vagal trunks at the level of the esophageal hiatus in the abdomen. There have been two randomized controlled trials of VBLOC therapy, the Empower study and the ReCharge study. In the Empower study, 294 subjects were implanted with the vagal electrodes and randomized to the treated (*n* = 192) or control (*n* = 102) group (Sarr et al. 2012). Main outcome measures were percent excess weight loss (EWL) at 12 months and serious adverse events. While the primary end point of EWL in the treatment group was not achieved, it was found that weight loss was related linearly to hours of device use; treated and controls with ≥12 h/day use achieved 30 ± 4 and 22 ± 8% EWL, respectively. It was concluded that the VBLOC® device therapy to treat morbid obesity was safe, but weight loss was not statistically greater in treated group compared to the control group. However, clinically important weight loss was related to the number of hours that the device was used. In the ReCharge study, one hundred sixty-two patients received an active vagal nerve neuromodulation device and 77 received a sham device (Ikramuddin et al. 2014). In the intent-to-treat analysis, the vagal nerve block group had a mean 24.4% excess weight loss (9.2% of their initial body weight loss) vs 15.9% excess weight loss (6.0% initial body weight loss) in the sham group. The mean difference in the percentage of the excess weight loss between groups was 8.5 percentage points (95% CI, 3.1–13.9), which did not meet the primary endpoint. However, weight loss was statistically greater in the vagal nerve block group. Apovian et al. reported the results of the ReCharge trial at 24 months, 123 (76%) vBloc participants remained in the trial (Apovian et al. 2017). Participants who presented at 24 months (*n* = 103) had a mean excess weight loss (EWL) of 21% (8% total weight loss [TWL]); 58% of participants had ≥5% TWL and 34% had ≥10% TWL. Among the subset of participants with abnormal preoperative values, significant improvements were observed in mean LDL and HDL cholesterol, triglycerides, HbA1c, and systolic and diastolic blood pressures. Heartburn/dyspepsia and implant site pain were the most frequently reported adverse events. The primary related serious adverse event rate was 4.3%. It was concluded that vBloc therapy continues to result in medically meaningful weight loss with a favorable safety profile through 2 years. Finally, Huerta et al. has reported the use of ultrasound stimulation at the portahepatis in the mice model of obesity (Huerta et al. 2021). Their results indicate that daily ultrasound stimulation for 8 weeks significantly decreased the body weight, and improved circulating lipids.

## Conclusion

Vagus nerve is a promising bioelectronic target for many chronic disorders; however, precision neuromodulation is needed to expand the horizon of the VNS application. Considering its anatomical structure, the vagus nerve can be stimulated at three different locations. First, at the cervical level, developing the nerve-electrode interface that preferentially stimulates a part of the fascicular structure, and stimulation strategies that can engage specific fiber types will give rise to the possibility of precision VNS that targets specific functions. Second, an electrode implanted on the individual branch that is near the target organ to effectively modulate specific neural signaling pathways. Third, at the organ level with the use of non-invasive ultrasound. We believe the precision VNS can be achieved through one of the above strategies, and the optimal approach should be highly disease-oriented and anatomically driven.

## Data Availability

Not applicable.

## Abbreviations

VN: Vagus nerve
VNS: Vagus nerve stimulation
cVN: Cervical vagus nerve
pVNS: Precision vagus nerve stimulation
aVNS: Auricular vagus nerve stimulation
nVNS: Non-invasive vagus nerve stimulation
FUS: Focused ultrasound stimulation
KES: Kilohertz electrical stimulation
SAP: Systemic arterial pressure
HR: Heart rate
BR: Breathing rate
